# Effect of varying levels of formaldehyde treatment of mustard oil cake on rumen fermentation, digestibility in wheat straw based total mixed diets *in vitro*

**DOI:** 10.14202/vetworld.2015.551-555

**Published:** 2015-04-29

**Authors:** Mahima, Vinod Kumar, S. K. Tomar, Debashis Roy, Muneendra Kumar

**Affiliations:** 1 Department of Animal Nutrition, College of Veterinary Science and Animal Husbandry, Uttar Pradesh Pandit Deen Dayal Upadhayay Pashu Chikitsa Vigyan Vishwavidyalay Evum Go-Anusandhan Sansthan (DUVASU), Mathura, Uttar Pradesh, India; 2 Dairy Cattle Nutrition Division, National Dairy Research Institute, Karnal, Haryana, India

**Keywords:** ammonia release, *in vitro* gas fermentation technique, mustard cake, ruminal fluid, ruminal fermentation, protected protein, protein fractions, volatile fatty acids

## Abstract

**Aim:**

The aim of the current study was to protect the protein in mustard cake by different levels of formaldehyde treatment with a view to optimize the level of formaldehyde.

**Materials and Methods:**

Different levels of formaldehyde treatment (0, 1, 1.5 and 2% of crude protein) containing concentrate and roughages diet in 40:60 ratio were tested for their effect on nutrients digestibility, *in vitro* ammonia release, *in vitro* gas production and change in protein fractions. Non-significant (p≤0.05) effect on pH, microbial biomass, partitioning factor, total gas production (TGP), TGP per g dry matter and TGP per g digestible dry matter (ml/g) was observed in almost all the treatments.

**Results:**

Total volatile fatty acids at 2% formaldehyde treatment level of mustard cake was lower (p<0.05) as compared to other groups, while *in vitro* dry matter digestibility and *in vitro* organic matter digestibility were reported to be low in 1% formaldehyde treated group.

**Conclusion:**

On a holistic view, it could be considered that formaldehyde treatment at 1.5% level was optimal for protection of mustard oil cake protein.

## Introduction

In most of developing countries including India, agriculture by-products, crop residues and grazing along with some protein and energy supplements are the chief feed source for livestock of major feed resource for ruminant livestock. The common protein supplements for ruminants are oil seed cakes obtained as a by-product of the oil industry. Among them, mustard cake is the most commonly available protein supplement for livestock in northern parts of India [1,2]. However, the protein value of mustard cake is low due to high rumen degradability [3]. The levels of rumen degradable protein (RDP) and rumen undegradable protein (RUP) of mustard oil cake is reported as 33% and 4%, respectively, hence protection of mustard cake protein assumes significant importance. There are reports that the efficiency of nitrogen utilization can be optimized/increased by manipulating the composition of RDP and RUP proportions [4,5]. The rapid and high rate of rumen degradation of rapeseed cake leads to excessive amount of ammonia production in the rumen, some of the ammonia produced in the rumen is utilized by the rumen bacteria for microbial protein synthesis. The remaining ammonia is either recycled and Wor excreted as waste. This rapid and high degradation of mustard cake protein in the rumen signifies the need of research to protect the protein from degradation and to minimize the wastage of valuable protein. Different approaches *viz*., heat [6] and chemicals including formaldehyde [7], alcohol [8], tannins [9], and sodium hydroxide [10] have been tried to protect the proteins from microbial degradation [11]. Among these methods, formaldehyde treatment of cake is the most common, efficient and comparatively cheaper method used to protect protein from microbial degradation in the rumen [7] and supplying good amino acid profile to the lower tract.

In the present study, an attempt was made to protect the protein in mustard cake by different levels of formaldehyde treatment with a view to optimize the level of formaldehyde. In a successful protection method, the protein should remain undegradable in the rumen and available in the intestine for absorption. Different levels of formaldehyde treatment were evaluated in terms of nutrients digestibility, *in vitro* ammonia release, *in vitro* gas production and change in protein fractions.

## Materials and Methods

### Ethical approval

Permission of taking animals for this experiment was duly taken from Institutional Animal Ethics committee (IAEC) constituted as per the article no. 13 of the CPCSEA rules laid down by Government of India.

### Feeds and experimental design

To evaluate the effect of different levels of formaldehyde treatment, diets were prepared by taking roughages and concentrate in the ratio of 60:40 and milled to pass through 1 mm seive. The roughages part composed of wheat straw and the concentrate part composed of barley (55%), wheat bran (10%), mustard cake (33%) and mineral mixture (2%) (Table-1). Mustard cake used for the preparation of concentrate was untreated, treated with formaldehyde without mineral mixture and treated with formaldehyde and mineral mixture. Mineral mixture was added at the ratio that the final concentration in prepared diet was 0.8 parts. A set was incubated devoid of substrate which served as blanks for particular treatment and control.

**Table-1:** Composition of experimental mineral mixture (with salt).

Ingredients	Percent
Moisture	5.0
Calcium	18.0
Phosphorus	9.0
Magnesium	5.0
Salt	22.0
Iron	0.4
Iodine as (KI)	0.02
Copper	0.06
Maganese	0.10
Cobalt	0.09
Fluorine	0.05
Zinc	0.30
Sulfur	0.40
Acid insoluble ash	3.00

### Formaldehyde treatment

Formaldehyde treatment for protection of mustard cake was done by the standard method [12]. Briefly, the formaldehyde solution (40% w/v) was sprayed on the ground mustard cake to have a final concentration of 1.0, 1.5 and 2.0 per 100 g of crude protein of mustard cake. The sprayed mustard cake samples were mixed thoroughly and stored in plastic bags and then air-tight plastic boxes for the completion of the reaction [13]. Containers kept closed for 10 days after that opened the boxes and mustard cake was poured onto plastic sheets about 4 cm thickness in open space and allowed to air equilibrate for 72 h, then stored in air-tight plastic boxes until use.

### Collection of rumen liquor and preparation of inoculum

The rumen liquor of about 3 freshly slaughtered buffalo was collected from local slaughter house and pooled into an insulated air tight flask and brought into the laboratory. The rumen liquor was strained through four layers of muslin cloth in a glass flask, then the required amount of strained rumen liquor used as inoculum. Carbon dioxide gas was passed through the rumen liquor and maintained at 39±1.0°C temperature for use of preparation of inoculum. The incubation medium was prepared by standard protocol [14]. The experiment was conducted in triplicate. A 200±0.5 mg substrate was weighed and taken in glass syringes of 100 ml capacity. Sample was put on the bottom with the help of weighing boat with removable stem. The 30 ml incubation medium was dispensed anaerobically in each prewarmed (39°C) syringe. Plungers of syringes were greased with paraffin soft jelly for smooth movement and to prevent any leakage. Syringes were closed using clamps and recorded the volume of the mixture in the syringes and syringes were placed vertically in a wooden stand with hole to hold the syringes upright in the incubator ventilated by fan assisted forced air circulation at 39±0.5°C for 48 h.

### *In vitro* dry matter (DM) and organic matter (OM) degradability

DM degradability and OM degradability of feed sample in each syringe containing residue after incubation was estimated [15].

### Partitioning factor (PF) and microbial biomass (MBM) yield

The PF is calculated as the ratio of substrate truly degraded *in vitro* (mg) to the volume of gas (ml) formed by it. The MBM yield was calculated using the degradability of substrate and gas volume and stoichiometrical factor [16].

MBM (mg) = Total digestible OM (mg) - (gas volume × 2.25)

### *In vitro* rumen fermentation parameters

Total volatile fatty acid (TVFA) concentration (mmol/dl) in the supernatant was estimated [17]. Ammonia nitrogen (NH_3_-N) was estimated using supernatant of each syringe. A total of 1 ml of 2% alcoholic boric acid solution was taken in the inner compartment of conway cell. 1 ml of strained rumen liquor transferred in the outer compartment of conway cell and 1 ml of 50% potassium carbonate placed opposite to the rumen liquor sample in the outer compartment of the conway cell. Lid of conway cell immediately closed. The conway cell rotated 4-5 times and incubate at 39-40°C for 2 h in the incubator. The contents of the inner compartment were titrated against N/100 sulfuric acid.

### Total gas production (TGP)

After 48 h of incubation, TGP was estimated by the displacement of the piston by the gas produced. The gas produced due to fermentation of substrate was calculated by subtracting gas produced in blank syringe (containing no substrate, but only the inoculum and buffer) from total gas produced in the syringe containing substrate and inoculum and buffer.

### TGP ml per g DM (ml/g)


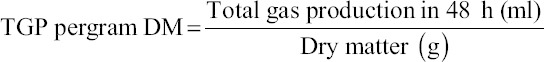


### TGP ml per g digestible DM (TGP/DDM)


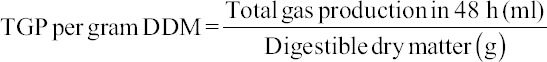


### Gas production at different incubation period

During the incubation, gas production was recorded at different intervals i.e., 0, 2, 4, 8, 12, 24, 36 and 48 h.

### Analysis of proximate principles and cell wall constituents

The proximate analysis of substrate was carried out as per standard protocol [18]. The neutral detergent fiber content of substrate was determined [15].

### Statistical analysis

Data obtained were processed to analysis of variance as per standard procedure [19] and means and homogenous subsets were separated using Duncans multiple range test [20].

## Results and Discussion

### Chemical composition of feed

In the present study, the concentrate and straw used in the ratio of 40:60 (concentrate: roughage). The chemical composition of concentrate and straw used in the *in vitro* study are presented in Table-2. The chemical composition of concentrate and straw was in agreement with the earlier reports [21-26].

**Table-2:** Chemical composition of feed ingredients (% DM basis) during *in vitro* trial.

Parameters	Concentrate	Wheat straw
DM	92.19	92.22
OM	87.88	90.24
Crude fiber	17.12	46.44
Ether extract	4.93	0.95
Crude protein	17.96	2.34
Nitrogen free extract	47.87	40.52
Nutrient detergent fiber	43.60	68.19
Acid detergent fiber	29.40	43.40
Acid detergent lignin	6.80	8.70
Cellulose	22.60	34.70
Hemicellulose	14.20	24.74

OM=Organic matter, DM=Dry matter

### pH, MBM and PF

Results of pH, MBM and PF *in vitro* study are given in Table-3. From the Table-3, it can be seen that the pH was relatively stable at near range (6.50-6.63). There was a nonsignificant difference in pH, MBM and PF in different treatments. The values of MBM and PF are in agreement with the previous report [27].

**Table-3:** Effect of different levels of formaldehyde treated mustard cake on *in vitro* parameters.

Parameters	Control	T_1_	T_2_	T_3_	T_4_	T_5_	T_6_	SEM	p value
pH	6.66	6.63	6.63	6.63	6.50	6.53	6.50	0.01	0.06
Microbial biomass (mg)	37.11	42.24	42.28	35.91	26.50	37.23	30.31	1.82	0.16
Partitioning factor	3.39	3.67	3.77	3.50	3.35	3.54	3.25	0.07	0.54
Gas production (ml/48 h)	33.00	29.83	27.66	28.50	25.33	30.00	30.50	0.74	0.15
TGP/g DM	165.00	149.16	138.33	142.50	126.66	150.22	152.50	3.73	0.15
TGP/g DDM	297.54	273.09	264.66	286.71	300.79	286.70	308.28	6.22	0.56
IVOMD%	55.68^c^	54.68^bc^	41.75^a^	52.27^bc^	52.37^bc^	50.02^bc^	49.47^b^	1.11	0.002
IVDMD%	53.80^c^	53.28^c^	36.43^a^	48.55^bc^	49.82^bc^	47.17^bc^	44.52^b^	1.48	0.006
TVFA (mmol/dl)	13.73^ab^	14.00^ab^	15.33^b^	15.20^b^	15.53^b^	11.40^a^	11.27^a^	0.47	0.01
Ammonia nitrogen (mg/dl)	14.00^c^	9.33^ab^	10.73^b^	8.40^ab^	7.47^a^	7.93^a^	8.40 ^ab^	0.53	0.001

Mean bearing different superscripts in a row differed significantly (p<0.05), *T_1_-1% HCHO treated mustard cake incorporated concentrate mixture, *T_2_-1% HCHO treated with mineral mixture mustard cake incorporated concentrate mixture, T_3_-1.5% HCHO treated mustard cake incorporated concentrate mixture, T_4_-1.5% HCHO treated with mineral mixture mustard cake incorporated concentrate, T_5_-2% HCHO treated mustard cake incorporated concentrate mixture, T_6_-2% HCHO treated with mineral mixture mustard cake incorporated concentrate mixture, IVDMD=*In vitro* dry matter digestible, IVOMD=*In vitro* organic matter digestible, DDM=Dry matter digestible, TGP=Total gas production

### Gas production

The gas production (ml) at different intervals (0, 2, 4, 8, 12, 24, 36 and 48 h) of incubation is presented in Table-3. The TGP ml, TGP per g DM (TGDM, ml/g) and TGP per g DDM (ml/g DDM) is presented in Table-3. There was no significant difference in TGP, TGDM and TGP per g DDM (TGDDM, ml/g DDM) at different levels of formaldehyde treatment. These results were similar to other values as reported previously [27].

### *In vitro* digestibility

Effect of variable concentration of formaldehyde used for treatment of mustard cake on *in vitro* dry matter digestibility (%) and OM digestibility (%) are presented in Table-3. Results of DM digestibility of composite diet found to be not affected by formaldehyde treatment. The results were nonsignificant among different treatments due to different concentrations of formaldehyde. There was also no adverse effect on digestibility. These results are in agreement with the results previously reported by other workers using different [27].

### *In vitro* rumen fermentation parameters

The effect on *in vitro* TVFA (mmol/dl RL) and ammonia nitrogen (NH_3_-N, mg/dl of rumen liquor) are given in Table-3. TVFA values in group T_1_, T_2_, T_3_ and T_4_ were higher but the difference was not significant, while TVFA level in group T_5_ and T_6_ was lower (p<0.05) as compared to control and T_1_ to T_4_. Lower level of TVFA in the T_5_ and T_6_ groups could be due to over-protection of protein (2% formaldehyde treated group) in the diet of these groups and might not have provided a favorable ratio of degraded and undegraded protein. Increased TVFA content in formalin preserved maize group have also been reported earlier [28,29].

The ammonia nitrogen (NH_3_N) levels were statistically low (p<0.05) in group T_1_ and T_2_ than control but lowest (p<0.01) in group T_3_, T_4_, T_5_ and T_6_ as compared to control. The ammonia nitrogen (mg/dl) values were lower in formaldehyde treated group than the control and lowest (p<0.01) level was in group T_3_, T_4_, T_5_ and T_6_. Similarly, lower (p<0.01) ammonia level at 1% formaldehyde treatment was reported [29] but contrary to our findings there was no statistically significant (p<0.01) reduction in ammonia production was observed on increasing the level of formaldehyde beyond 1%, while decreased level of NH_3_N with increased level of formaldehyde treatment of barley grain was reported [30]. Present findings were also supported by earlier studies [31,32] that ammonia production was inhibited whenever the soybean meal was treated with 1.5 and 3.0% formaldehyde. Rumen ammonia concentrations remain low with increasing level of protein until maximal microbial production is attained.

## Conclusion

It can be deduced that 1.5% formaldehyde treatment to mustard oil cake will be beneficial in increasing digestible undegradable protein in wheat straw containing diet without affecting the digestibility, but more studies are needed to validate the results under *in vivo* conditions. The result of this study might have implication in all other developing countries where the diet is based on straw and mustard cake, which is highly degradable in the rumen. Thus, protection of mustard cake by formaldehyde treatment may help in improving the protein supplementation of ruminants.

## Authors’ Contributions

M, VK and SKT have conceived, planned and designed the study. M and DR recorded and analyzed the data. MK provided technical support. Manuscript was drafted and revised by M, VK and DR under the guidance of SKT. All authors read and approved the final manuscript.
